# Solvent Effects on Morphology and Electrical Properties of Poly(3-hexylthiophene) Electrospun Nanofibers

**DOI:** 10.3390/polym11091501

**Published:** 2019-09-14

**Authors:** Jung-Yao Chen, Chien-You Su, Chau-Hsien Hsu, Yi-Hua Zhang, Qin-Cheng Zhang, Chia-Ling Chang, Chi-Chung Hua, Wen-Chang Chen

**Affiliations:** 1 Department of Chemical Engineering, National Chung Cheng University, Chiayi 62102, Taiwan; roorroorroorroor@hotmail.com (C.-Y.S.); orz520r1995@gmail.com (C.-H.H.); a0972795820@gmail.com (Y.-H.Z.); velevelev1@yahoo.com.tw (Q.-C.Z.); df89003485@yahoo.com.tw (C.-L.C.); 2 Department and f Chemical Engineering, National Taiwan University, Taipei 10617, Taiwan

**Keywords:** electrospinning, conjugated polymer, solvent effect, organic field-effect transistor

## Abstract

Herein, poly(3-hexylthiophene-2,5-diyl) (P3HT) nanofiber-based organic field-effect transistors were successfully prepared by coaxial electrospinning technique with P3HT as the core polymer and poly(methyl methacrylate) (PMMA) as the shell polymer, followed by extraction of PMMA. Three different solvents for the core polymer, including chloroform, chlorobenzene and 1,2,4-trichlorobenzene, were employed to manipulate the morphologies and electrical properties of P3HT electrospun nanofibers. Through the analyses from dynamic light scattering of P3HT solutions, polarized photoluminescence and X-ray diffraction pattern of P3HT electrospun nanofibers, it is revealed that the P3HT electrospun nanofiber prepared from the chloroform system displays a low crystallinity but highly oriented crystalline grains due to the dominant population of isolated-chain species in solution that greatly facilitates P3HT chain stretching during electrospinning. The resulting high charge-carrier mobility of 3.57 × 10^−1^ cm^2^·V^−1^·s^−1^ and decent mechanical deformation up to a strain of 80% make the P3HT electrospun nanofiber a promising means for fabricating stretchable optoelectronic devices.

## 1. Introduction

Conjugated polymer-based organic field-effect transistors (OFETs) have received considerable attention because of their solution-processable and flexible features [1,2]. Intensive research toward OFETs has shown that the microstructure and electrical property of poly(3-hexylthiophene) (P3HT) are critically affected by parameters during the solution process such as the processing solvent [3,4,5], annealing temperature and surface hydrophobicity of the substrate [6,7,8].

Among the parameters during the solution process, the processing solvent plays a critical role during thin-film formation due to the variation of chain conformation in the pristine solution [9,10]. It has been reported that P3HT chains preferentially adopt an edge-on orientation with π−π stacking parallel to the substrate, which allows the holes hopping between polymer chains to be the main transport mechanism in P3HT thin films. Often, the use of a low boiling point solvent and the consequently rapid solvent evaporation limit the time for crystallization during a spin-coating process and thus inhibit the charge-carrier transportation through π−π stacking [3,4,11].

Other studies suggest that the orientation of crystalline grains can also play a critical role in dictating the charge-carrier transportation. Specifically, highly-orientated crystalline grains with respect to the substrate can serve as an effective charge transport conduit for the charge-carriers without interfering with the hopping along the π−π stacking [12]. In addition, conjugated polymer films with a high crystallinity will render them stiff and brittle and thus limit the application on stretchable electronics [13,14]. Therefore, the (bulk) orientation of crystalline grains and the (local) degree of crystallinity can both affect the field-effect mobility of P3HT thin films while determining their mechanical property.

**Figure 1:**
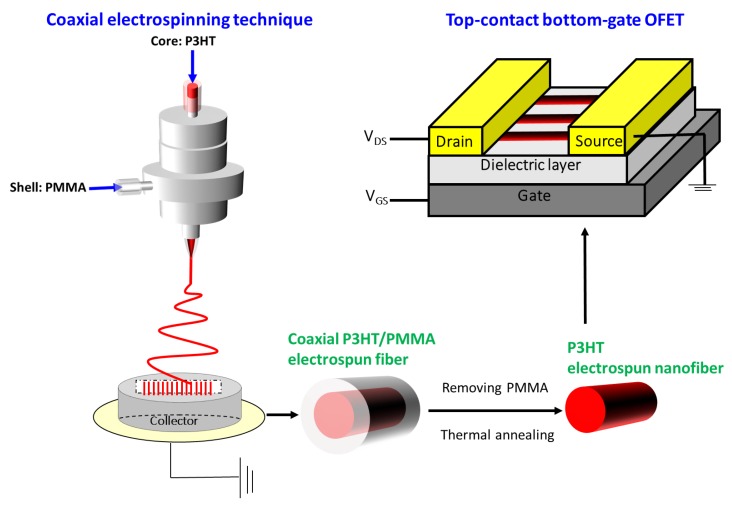
Schematic of the coaxial electrospinning setup and poly(3-hexylthiophene-2,5-diyl) (P3HT) electrospun nanofiber-based organic field-effect transistors (OFETs). Abbreviations: PMMA, poly(methyl methacrylate).

In this report, we prepared P3HT electrospun nanofiber-based OFETs by a coaxial electrospinning technique. The nanofibers consist of a P3HT core and poly(methyl methacrylate) (PMMA) shell, followed by a solvent extraction of PMMA, as schematically illustrated in Figure 1. Three different solvents for the core polymer including chloroform, chlorobenzene and 1,2,4-trichlorobenzene were utilized to manipulate the P3HT solution properties. We investigated the solvent-regulated, morphological and structural features of the P3HT electrospun nanofibers through dynamic light scattering (DLS), scanning electronic microscopy (SEM), X-ray diffraction (XRD) and photoluminescence (PL) characterizations, as well as their influences on the ductility of P3HT electrospun nanofibers and the corresponding charge-carrier mobility for OFETs. Our experimental results and analyses indicate that a higher solubility in the chloroform system greatly facilitates P3HT chain stretching along the long axis of the nanofiber during electrospinning, which in turn enables the formation of highly oriented crystalline grains after thermal annealing and leads to improvement both on the ductility of P3HT electrospun nanofibers and the performance of P3HT electrospun nanofiber-based OFETs.

## 2. Materials and Methods 

Materials: poly(methyl methacrylate) (PMMA), octadecyltrichlorosilane (ODTS), chloroform (CF, anhydrous ≥99%), chlorobenzene (CB, anhydrous 99.8%,) and 1,2,4-trichlorobenzene (TCB, anhydrous ≥99%) were purchased from Sigma-Aldrich (Milwaukee, WI, USA). Tetrabutylammonium perchlorate (TBAP, TCI (Tokyo, Japan)) was used as received without further purification. Poly(3-hexylthiophene) (P3HT) (*M*_w_ ~50,000, 90–95% regioregular) was provided by Rieke Metals Inc (Lincoln, NE, USA).

Electrospinning process: A coaxial electrospinning technique was used to produce core-shell electrospun fibers, similar to that reported in the literature [15,16,17,18,19,20]. Figure 1 shows a schematic illustration of our experimental setup. As shown in Figure 1, two syringes containing a P3HT core and PMMA shell solutions were connected through the coaxial spinneret to form a core-shell electrospinning system. Then, 300 mg·mL^−1^ of PMMA was dissolved in chlorobenzene with 10 wt % of TBAP as the shell solution to increase conductivity and stabilize the cone-jet. Next, 50 mg·mL^−1^ of P3HT as the core solution was dissolved in anhydrous CF, CB and TCB, respectively under an N_2_-filled glove box. The two solutions were fed into the coaxial spinneret by two syringe pumps (KD Scientific Model 100, Holliston, MA, USA) with the feed rate fixed at 0.1 and 1.0 mL·h^−1^ for P3HT core solution and PMMA shell solution, respectively. A high-voltage power supply (chargemaster CH30P SIMCO, Hatfield, PA, USA) set at 9–10.7 kV was connected to the tip of the core needle. The aligned electrospun fibers were collected by two parallel metal wires (~3 cm apart) below the tip of the needle, similar to that reported in the literature [21,22,23]. The coaxial electrospinning process was carried out under an ambient environment.

Device fabrication: To reduce the surface trapping site and immobilize the P3HT electrospun nanofibers during solvent washing, ODTS self-assembling monolayer was grafted on heavily n-type doped wafers with 200 nm thick SiO_2._ Aligned coaxial P3HT/PMMA electrospun fibers collected from the above two parallel metal wires were transferred to the silicon wafer. To remove the PMMA shell, aligned core-shell electrospun fibers on wafers were immersed in acetone for 1 h. After drying, thermal annealing at 100 °C was exempted for 20 min to increase the crystallinity of P3HT. The top-contact and bottom-gate organic field-effect transistor (OFET) with a channel length (L) of 25 μm and width (W) of 1500 μm was defined by thermal deposited gold through a shadow mask.

Ductility test: A polydimethylsiloxane (PDMS, Sylgard 184, Dow Corning, Midland, MI, USA) substrate (~2 mm thick) was prepared at a ratio of 10:1 (base: crosslinker, *w*/*w*) and cured for 12 h at 80 °C. Carbon nanotubes (CNTs) in CF at a concentration of 0.2 mg·mL^−1^ which contain 25 wt % of P3HT to homogeneously disperse bundled CNTs were ultrasonicated for 60 min. CNTs were first spray-coated onto a PDMS through a shadow mask with a gap distance of 300 μm. Highly aligned coaxial P3HT/PMMA fibers were transferred from the collector to the above CNT/PDMS substrate and then acetone was sprayed on them to remove the PMMA shell.

Characterization: Dynamic light scattering (DLS) measurements were conducted using a laboratory-built apparatus that is equipped with a 34 mW polarized He−Ne laser ($\lambda_{0}$ = 632.8 nm; Lasos, LGK 7626S, Jena, Germany), as described elsewhere [24,25]. To minimize the impact of multiple scattering while producing fair-quality DLS data, all characterizations were performed on 1 mg·mL^−1^ P3HT solutions to gain insight into the solvent-regulated, pristine aggregation properties. All of the sample solutions were filtered through a 0.45 μm polytetrafluoroethylene (PTFE) Millipore filter to remove dust before the measurement. The sample solution, 1 mL in volume, was loaded into a cylindrical quartz cell 1 cm in diameter (Hellma, 540.111, Müllheim, Germany), and the cell was carefully cap-sealed to prevent solvent evaporation during the entire experiment. All measurements were conducted at 25.0 ± 0.1 °C for a range of scattering angles θ = 30°−90°. Details of the analysis scheme can be found in prior work [25,26]. Viscosities of solutions were determined by AR2000ex system (stress-controlled) and TA Instruments at room temperature with stainless steel, 40 mm, 4 deg cone. The external structure of the prepared ES fibers was characterized by a field-emission scanning electron microscope operated at an accelerating voltage of 10 kV. Before imaging, the samples were sputtered with platinum. Optical characteristics of P3HT electrospun nanofibers were detected by UV–Vis spectrophotometer (Hitachi U-4100, Tokyo, Japan) and polarized steady-state photoluminescence (PL) (Horiba Jobin Yvon, Kyoto, Japan). Transmission-mode wide angle X-ray scattering (WAXS) and two-dimensional grazing incidence X-ray diffraction (2D-GIXRD) at beamline stations 17A1 and 13 A1 of the National Synchrotron Radiation Research Center were used to investigate the internal molecular structure of P3HT electrospun nanofibers.

Electrical performance of the P3HT electrospun nanofibers-based OFET devices were characterized by Keithley 4200 semiconductor parametric analyzer (Beaverton, OR, USA). The relationship *I*_DS_ = μ(CL^−2^)·(*V*_GS_ − *V*_TH_)^2^ was used to obtain the charge-carrier mobility (µ) in the saturation region, where *I*_DS_ *, V*_GS_ and *V*_TH_ is drain current, gate voltage and threshold voltage, respectively. The capacitance per unit length (CL^−1^) is described by 2πεε*_0_*(ln(2hr^−1^))^−1^ with a dielectric layer thickness (*h*) of 200 nm, average dielectric constant (ε) of 2.5 and measured radius (*r*) of the P3HT electrospun nanofiber [27].

## 3. Results

### 3.1. Solution Rheology

**Figure 2:**
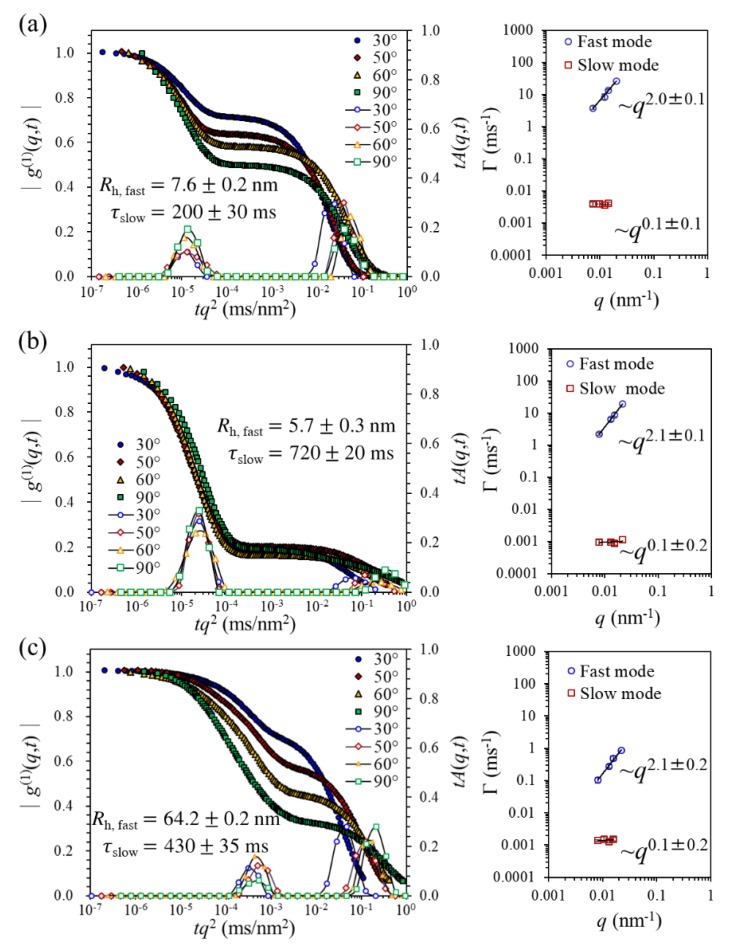
Angular dependences of the field autocorrelation function and the associated decay time distribution extracted from Constrained Regularization Method for Inverting Data (CONTIN) for 1 mg·mL^−1^ P3HT solutions prepared with (**a**) chloroform (CF); (**b**) chlorobenzene (CB) and (**c**) 1,2,4-trichlorobenzene (TCB) media, where the decay time *t* has been rescaled with *q*^2^. The right column shows the scaling relationship between the mean decay rate (〈Γ〉) and the scattering vector (*q*) (i.e., $\langle \Gamma \rangle \sim q^{\alpha }$), which can be utilized to identify the attribute of a particular relaxation mode (e.g., α = 2 for diffusive motions).

To reveal the intrinsic aggregation features of P3HT chains in different solvent systems, P3HT solutions were diluted to 1 mg·mL^−1^ for dynamic light scattering (DLS) analysis. The DLS results on the field autocorrelation function, $\left|g^{\left(1\right)}\left(q,t\right)\right|$, and the associated decay time distribution, A(q,t), for three dilute P3HT solutions are presented in Figure 2. The existence of two distinct relaxation modes can be readily identified in all cases, wherein the first (fast) mode exhibits the usual diffusive motion (i.e.,$\;\langle \Gamma \rangle \sim q^{2}$ ) while the second (slow) mode demonstrates a peculiar *q*-independence (i.e., $\langle \Gamma \rangle \sim q^{0}$). The hydrodynamic radius, $R_{h}$ , estimated from the Stokes–Einstein relation for the fast modes of CF and CB solutions ($R_{h}$ ~5–8 nm) indicates the dominance (about 99% in weight fraction, as evaluated by the intensity-weight average [26]) of isolated-chain species, whereas the TCB solution bears a substantially greater size ($R_{h}$ ~60 nm) that can be attributed to crystalline aggregates, as often reported on poorer-solvent media for P3HT [28,29]. The aggregate species clearly is unfavorable for controlling chain orientation in electrospun nanofibers, as we discuss later in the X-ray diffraction (XRD) and photoluminescence (PL) analyses. The *q*-independent slow mode, ubiquitous in all three P3HT solutions investigated, might reflect the contribution of nondiffusive, microsized clusters that undergo a dynamic condensation/decomposition alternating process [9]. Judged solely by the apparent relaxation time along with the solvent viscosity, the cluster size appears to follow the order: TCB < CF < CB. According to the implied solvent quality in the discussions above on the fast-mode properties, it is likely that the TCB solution produces a smaller, yet more compact, aggregate cluster when compared with the other two. Given, however, that the cluster species is estimated to constitute only about 1% in weight fraction, its impact on ES nanofibers remains unclear.

**Figure 3:**
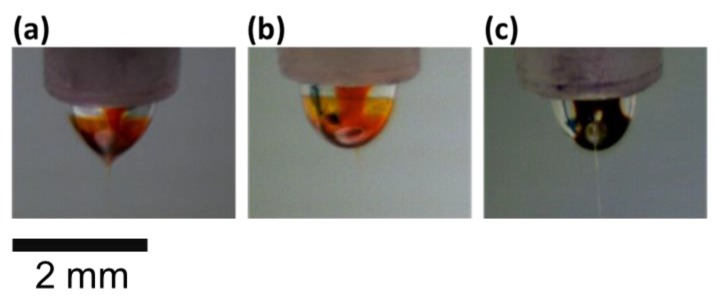
Charge-coupled Device (CCD) images of Taylor cone in (**a**) CF, (**b**) CB and (**c**) TCB systems.

To facilitate the comparison between different solvent systems, the physical properties of three solvents used in this study and those of the corresponding P3HT solutions are summarized in Table S1 (in Supplementary Materials). It is worth noting that the viscosities of P3HT solutions at a concentration of 50 mg·mL^−1^ are determined to be 6.22, 5.40 and 21.12 centipoise for CF, CB and TCB systems, respectively, as they represent an essential parameter during electrospinning. Figure 3 show the optical images of a coaxial nozzle during electrospinning for the three solvent systems, where P3HT shows notable color variations in response to the effect of processing solvent. CF and CB are two better solvents for P3HT and result in bright and transparent reddish-orange in the core. TCB is a poorer solvent and results in deep brown in the middle. When CF or CB is used as the solvent, the P3HT solution accumulates on the tip of the Taylor cone and even forms a thin film on the surface layer of the cone. In contrast, the diffusion of core P3HT solution to the outside layer of the cone was prohibited due to the high viscosity of the core solution in the TCB system.

### 3.2. Morpholgy of P3HT Electrospun Nanofibers

**Figure 4:**
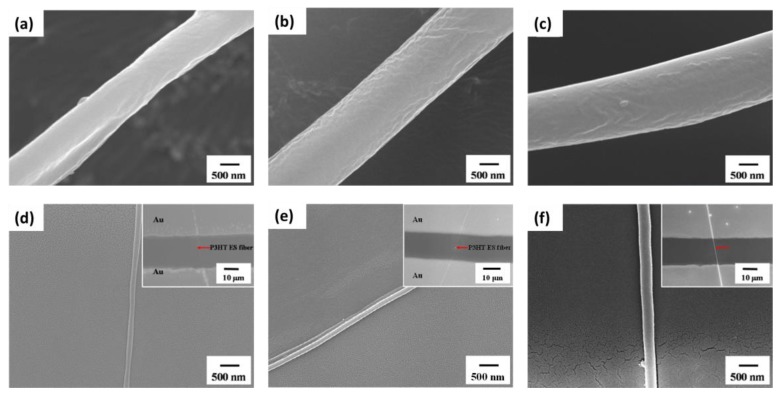
Scanning electronic microscopy (SEM) images of as-spun P3HT/PMMA fibers prepared from (**a**) CF; (**b**) CB and (**c**) TCB systems. The corresponding pure P3HT nanofibers prepared from (**d**) CF; (**e**) CB and (**f**) TCB systems after removing the PMMA shell. The inset SEM figures show the typical transistor fabricated by the respective P3HT nanofibers.

The SEM images shown in Figure 4 indicate that the as-spun coaxial P3HT/PMMA fibers prepared from three different solvents bear a similar diameter of about 1~2 μm. After the removal of the PMMA shell by immersing the fiber in acetone, all of the P3HT electrospun nanofibers are smooth and uniform in appearance because evaporation of the core solvent is prohibited by the shell material, leading to a more stable cone-jet model during the electrospinning process. The diameters of naked P3HT electrospun nanofibers are determined to be 141, 168 and 265 nm for CF, CB and TCB systems, respectively. The pronounced change in diameter for the P3HT electrospun nanofibers can be attributed to the varying degree of polymer aggregation which prohibits P3HT chain stretching during electrospinning.

**Figure 5:**
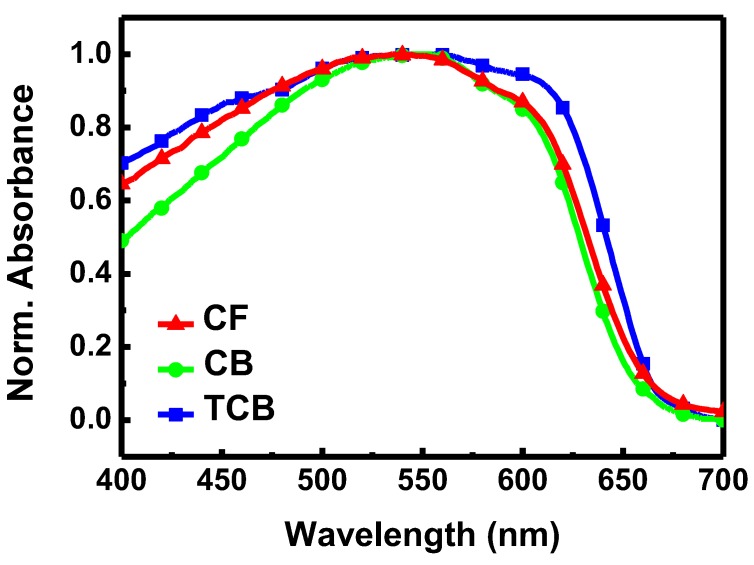
Optical absorption spectra of P3HT electrospun nanofibers prepared from three different solvent systems.

The UV–Vis absorption spectra of P3HT electrospun nanofibers prepared from the three solvent systems are shown in Figure 5. After normalization by the maximum absorption peak (at wavelength ~550 nm), the intensities of the shoulder (at wavelength ~600 nm) follow the order: CF < CB < TCB. The difference likely reflects the number of ordered aggregates associated with interchain π−π stacking of P3HT [30,31]. According to an early study by Hagler et al., the intensity increase of the shoulder originates from the increase of crystallinity [32]. A high crystallinity in the TCB system is probably due to the severe aggregation of P3HT in the solution, as resolved in prior DLS analysis.

**Figure 6:**
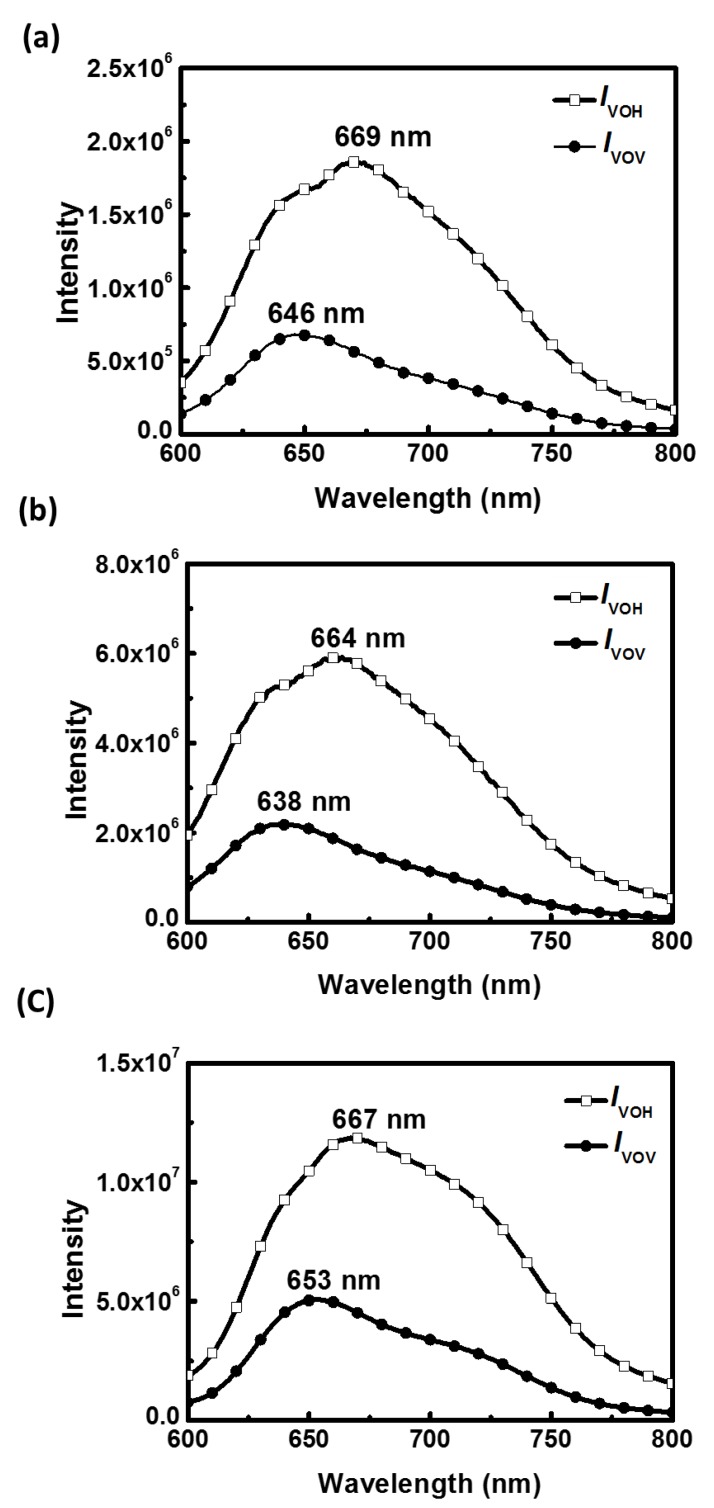
Polarized photoluminescence spectra of the aligned P3HT electrospun nanofibers, with an isotropic exciting light source and the corresponding emission collected for the perpendicular (*I*_VOH_) or parallel (*I*_VOV_) polarization with respect to the fiber axis for (**a**) CF; (**b**) CB and (**c**) TCB systems.

The notable impact of solvent systems for the core polymer can be further observed in polarized photoluminescence (PL) spectra of the aligned P3HT electrospun nanofibers, as shown in Figure 6. The emission polarizations perpendicular and parallel to the nanofiber axis were defined as *I*_VOH_ and *I*_VOV_, respectively. All PL spectra of aligned P3HT electrospun nanofibers prepared from different solvents show polarization anisotropy. When P3HT electrospun nanofibers were excited by an unpolarized light source at a wavelength of 550 nm, the PL polarization ratios (*I*_VOH_/*I*_VOV_) of 2.72, 2.69 and 2.34 are observed for CF, CB and TCB systems, respectively. In addition, the shifts of PL maximum emission peak between *I*_VOH_ and *I*_VOV_ are 23, 26 and 14 nm for CF, CB and TCB systems, respectively. The greater polarization ratios may be attributed to the extended P3HT chain conformation fostered in the CF and CB solutions which can better respond to the elongation force during electrospinning and lead to an enhanced orientation of lamellar spacing after thermal annealing. In contrast, the smallest shift of PL maximum emission peak between *I*_VOH_ and *I*_VOV_ in the TCB system indicates the lowest degree of orientation for P3HT chains in electrospun nanofibers.

**Figure 7:**
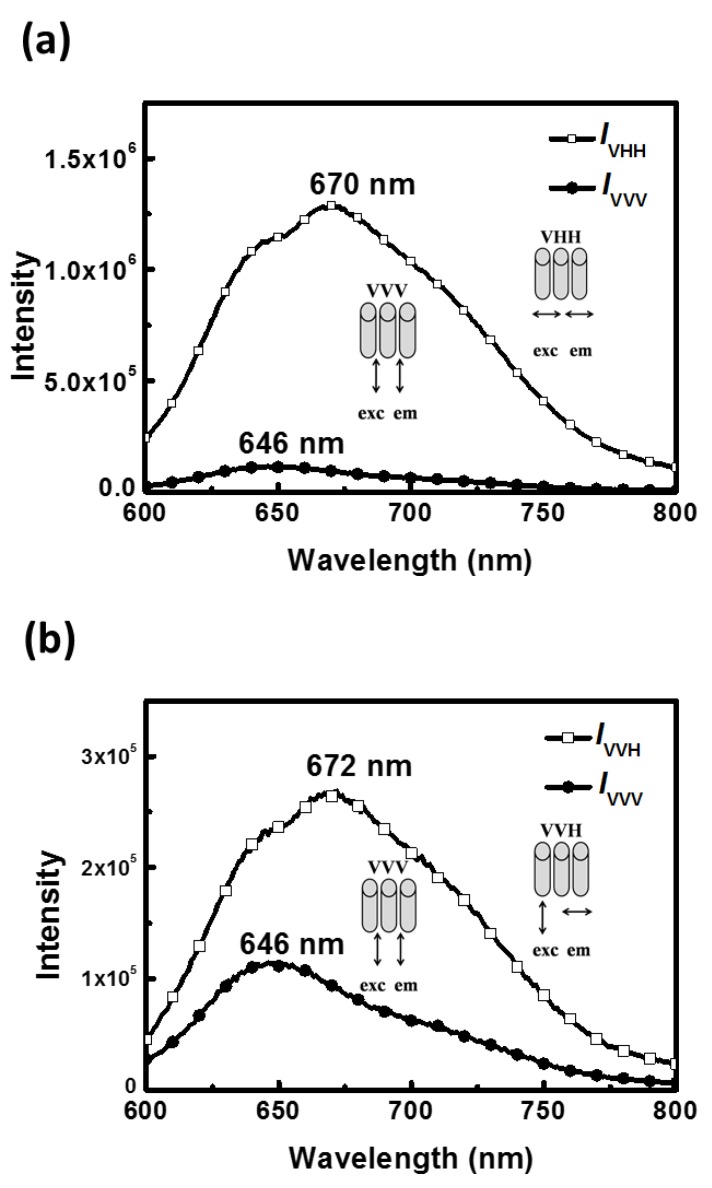
Polarized photoluminescence spectra of the aligned P3HT electrospun nanofibers prepared from the CF system with (**a**) the exciting light and the corresponding emission polarized in the perpendicular (*I*_VHH_) or parallel (*I*_VVV_) direction with respect to the fiber axis and (**b**) the exciting light polarized in the parallel direction with respect to the fiber axis, and the corresponding emission polarized in the perpendicular (*I*_VVH_) or parallel (*I*_VVV_) direction with respect to the fiber axis.

To further explore the P3HT chain orientation in the CF system, the polarized PL spectra of the P3HT electrospun nanofiber prepared from the CF system were systematically analyzed, as shown in Figure 7. As can be seen, the intensity and emission peak wavelength in the PL spectra respond to the polarizer direction significantly. All PL spectra with the emissions collected perpendicular to the nanofiber (main axis) are red-shifted in the maximum emission wavelength and show stronger intensity compared to the parallel ones regardless of whether the exciting light is parallel or perpendicular with the nanofiber. For example, the polarization ratio *I*_VHH_/*I*_VVV_ attains a value of 11.2 and the perpendicular one (λ = 670 nm) is red-shifted by 24 nm compared to that of the parallel one (λ = 646 nm) (Figure 7a). Interestingly, when the exciting light is polarized parallel to the nanofiber, more light is emitted that is polarized in the direction perpendicular to the nanofiber (Figure 7b). 

It has been known that energy can transfer rapidly via Förster transfer within a few picoseconds before emission. Therefore, emission is observed only from the longest, lowest-energy segments [33]. The above features suggest that excitons created on a shorter conjugation segment parallel to the nanofiber axis may migrate to a longer conjugation one that is perpendicular to the nanofiber axis, and then emit red-shifted and polarized light perpendicular to the nanofiber direction. Similar results concerning the perpendicular alignment of backbone have been reported in our previous work [34].

**Figure 8:**
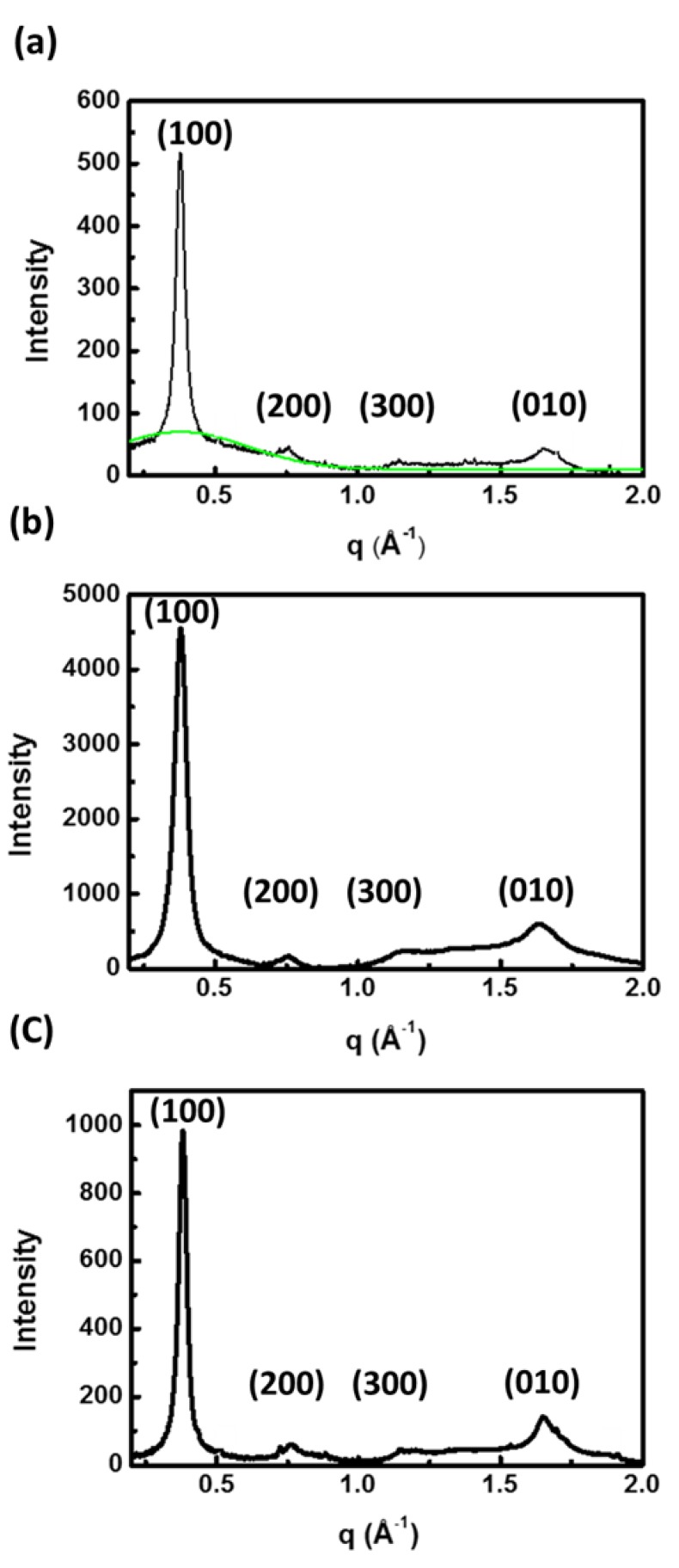
Transmission-mode X-ray diffraction patterns of nanofibers prepared from (**a**) CF, (**b**) CB and (**c**) TCB systems.

**Figure 9:**
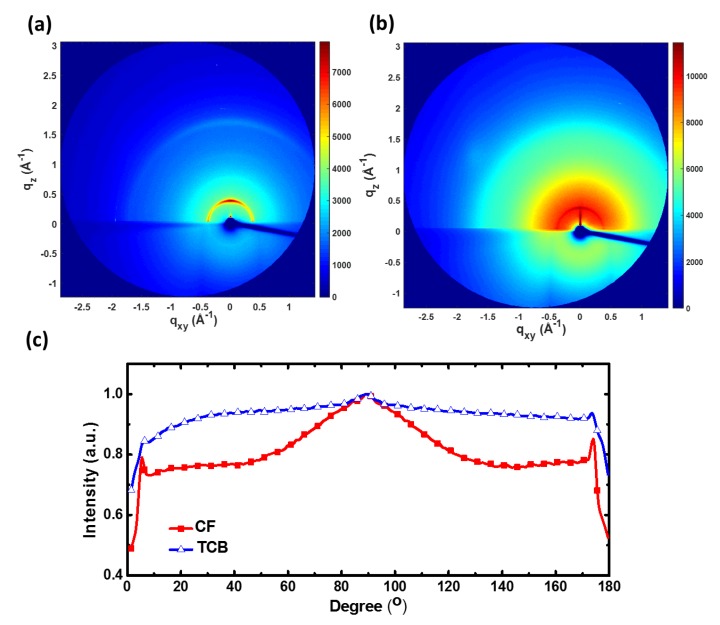
Two-dimensional grazing incidence X-ray diffraction (2D-GIXRD) patterns of P3HT electrospun nanofibers prepared from (**a**) CF and (**b**) TCB systems. (**c**) Azimuthal angle analysis of (100) lamellar packing for CF and TCB systems.

To measure the internal structure of P3HT electrospun nanofibers prepared from different solvent systems, wide-angle X-ray diffraction (WAXD) with transmission-mode was performed. In Figure 8, three ordered structures in the (100) direction labeled as (100), (200) and (300), respectively, which represent the lamella structure, arise from the interdigitation of the hexyl side chain, and the (010) reflection due to π–π interchain stacking is observed in all three solvent systems [34]. Note that P3HT nanofibers prepared from the TCB system have a sharp peak of (100), suggesting a high degree of P3HT crystallinity. It is interesting to note that in the CF system there is a broad amorphous halo under the peak of (100), which signifies the random packing of P3HT and a low degree of crystallinity [7]. Two-dimensional grazing incidence X-ray diffraction (2D-GIXRD) techniques, which measured across P3HT electrospun nanofibers, were employed to examine the orientation of P3HT chain packing in the nanofibers, as shown in Figure 9. The strong (100) diffraction in the *q*_z_ direction (azimuthal angle = 90°) in the CF system suggests that P3HT chains prevalently adopt an “edge-on” arrangement with respect to the fiber surface. For comparison, the broad azimuthal angle distribution observed for the TCB system is indicative of the random orientation of P3HT chain folding.

**Figure 10:**
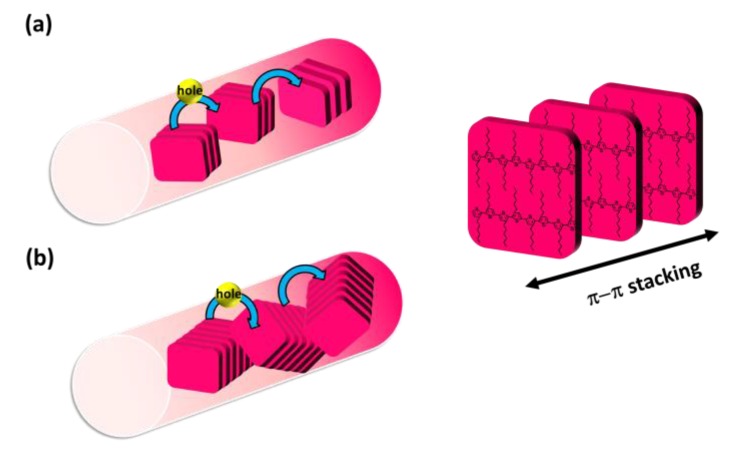
Schematic illustrations of the chain packing and crystalline grain orientation in P3HT electrospun nanofibers prepared from (**a**) CF or CB and (**b**) TCB systems, where the red bricks demonstrate the 2D planes from the thiophene rings.

According to the features discussed above, we conclude that the polymer aggregates fostered in the TCB solution, while contributing to increased ordering of alkyl chains and enlarged crystal size, lead to poor orientation between the crystalline grains in P3HT electrospun nanofibers, as illustrated in Figure 10b. In contrast, the extended polymer chains fostered in CF and CB solutions help produce highly orientated crystalline grains and smaller crystal size in P3HT electrospun nanofibers, as illustrated in Figure 10a.

### 3.3. Electrical Properties of P3HT Electrospun Nanofibers

**Figure 11:**
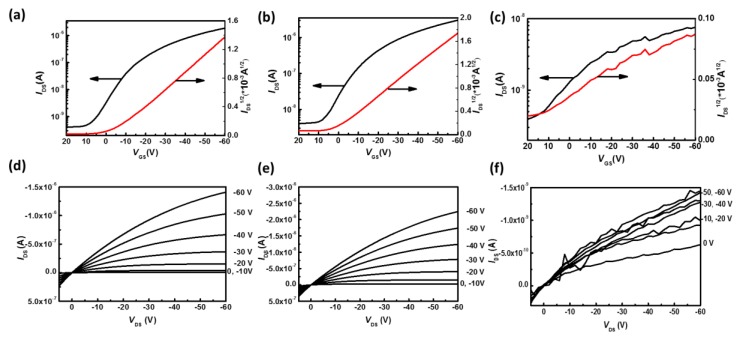
Transfer (*I*_DS_ and *I*_DS_^1/2^ versus *V*_GS_ at *V*_DS_ = −60 V) and output characteristics of P3HT electrospun nanofibers prepared from (**a**,**d**) CF; (**b**,**e**) CB and (**c**,**f**) TCB systems.

The transfer and output characteristics of P3HT electrospun nanofiber-based OFETs prepared from the three solvent systems are shown in Figure 11. All of the devices show field-effect characteristics; however, the *I*_DS_ seems to be unstable for the device prepared from the TCB system. This may be attributed to the poor orientation between crystalline grains in the nanofibers that impedes the hole hopping. Table 1 shows all the electrical properties of the devices mentioned above. From Table 1, the maximum mobilities for the three solvent systems are 3.57 × 10^−1^, 1.78 × 10^−1^ and 1.48 × 10^−4^ cm^2^·V^−1^·s^−1^, respectively. Intriguingly, the value of the charge-carrier mobility differs by as much as three orders of magnitude and appears to follow distinct trends as compared with those of the degree of crystallinity. The CF system shows the highest mobility despite the fact that its crystallinity remains the lowest, possibly because of the highly ordered orientation between crystalline grains that facilitates charge-carrier hopping through π–π stacking between polymer chains. Table S2 (in Supplementary Materials) summarizes the electrical performances of pure P3HT electrospun nanofiber-based organic field-effect transistors that have been reported in the literature. The results clearly reveal that the core-shell electrospinning technique and the effect of solvent play essential roles in producing high-performance P3HT electrospun nanofiber-based organic field-effect transistors.

### 3.4. Ductility Test of P3HT Electrospun Nanofibers

**Figure 12:**
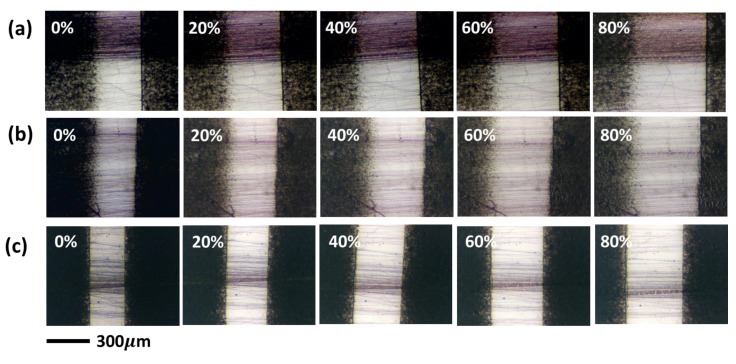
Ductility test of P3HT electrospun nanofibers prepared from (**a**) CF, (**b**) CB and (**c**) TCB systems. The red arrows indicate the crack formation.

**Figure 13:**
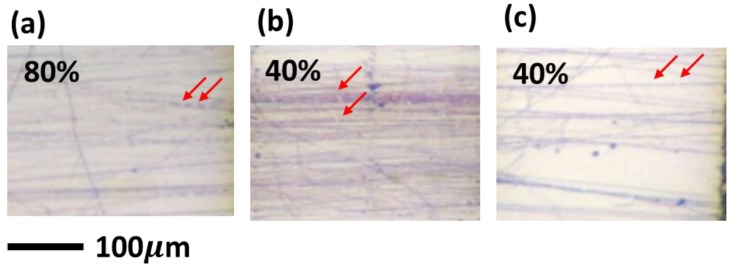
Enlarged optical images of crack formation for (**a**) CF, (**b**) CB and (**c**) TCB system. The red arrows demonstrate the location of crack formation.

To test the ductility of P3HT electrospun nanofibers, the nanofibers were deposited on polydimethylsiloxane (PDMS) substrate with two spray-coated carbon nanotube (CNT) mats as the gap markers. Figure 12 shows the optical images of the P3HT electrospun nanofibers prepared from different solvent systems when subjected to external strain. All of the aligned P3HT electrospun nanofibers deform along the stretching direction. However, when the strain is increased to 40%, crack formation can be clearly seen for the CB and TCB systems, as shown in Figure 13b,c. In contrast, P3HT electrospun nanofibers prepared from the CF system show superior ductility and crack formation appears only at a much higher strain of 80% (Figure 13a). The delayed crack formation for the CF system may result from the lower crystallinity as the amorphous part can better accommodate the external stress through deformation. Thus, when compared to the other two solvent systems, the CF system seems to achieve the best performance in producing P3HT electrospun nanofibers that possess simultaneously high charge-carrier mobility and decent mechanical ductility.

## 4. Conclusions

In this study, P3HT nanofiber-based OFETs were successfully prepared by a two-fluid coaxial electrospinning technique with P3HT as the core polymer and PMMA as the shell polymer, followed by extraction of PMMA. Three different solvents for the core polymer—CF, CB and TCB—were employed to manipulate the morphologies and electrical properties of P3HT electrospun nanofibers. The pronounced change in diameter for the P3HT electrospun nanofibers can be attributed to a relatively large viscosity of P3HT solution in the TCB system as a result of the prevalence of aggregate species in solution, which prohibits the diffusion of the core solution into the outside layer and the whipping process during electrospinning. In contrast, the CF system which fosters extended P3HT chains can be readily stretched by the electrical force, resulting in highly oriented crystalline grains and high charge-carrier mobility despite a lower crystallinity and smaller crystal size. The highest charge-carrier mobility thus achieved, 3.57 × 10^−1^ cm^2^·V^−1^·s^−1^, and the decent mechanical ductility of P3HT electrospun nanofibers fabricated from the CF system suggest the importance of chain stretching during the electrospinning process, whereby favorable orientation of crystalline grains in the electrospun nanofiber can be produced and contributes to excellent electrical properties and mechanical ductility at one time.

## Figures and Tables

**Table 1:** Diameters and electrical properties of the devices prepared from three different solvent systems.

Shell Flow Rate (ml·h^−1^)	Diameter (nm)	Mobility (cm^2^·V^−1^·s^−1^)	On Current (A)	Off Current (A)	On/Off (-)	V_TH_ (V)
CF	141	3.57 × 10^−1^	9.42 × 10^−7^	2.06 × 10^−10^	4.57 × 10^3^	−2.5
CB	168	1.78 × 10^−1^	4.33 × 10^−7^	5.74 × 10^−10^	7.54 × 10^2^	3.3
TCB	265	1.48 × 10^−4^	1.09 × 10^−9^	5.60 × 10^−11^	1.94 × 10^1^	40

## Supplementary Materials

The following are available online at https://www.mdpi.com/2073-4360/11/9/1501/s1, Table S1: physical properties of three solvents used in this study and those of the P3HT solutions, Table S2**:** state-of-the-art electrical performances of pure P3HT electrospun nanofiber-based organic field-effect transistors.

## References

[1] Klauk H. (2006). Organic Electronics: Materials, Manufacturing and Applications.

[2] Bao Z., Locklin J. (2007). Organic Field-Effect Transistors.

[3] Surin M., Leclere P., Lazzaroni R., Yuen J.D., Wang G., Moses D., Heeger A.J., Cho S., Lee K. (2006). Relationship between the microscopic morphology and the charge transport properties in poly(3-hexylthiophene) field-effect transistors. J. Appl. Phys..

[4] Chang J.-F., Sun B., Breiby D.W., Nielsen M.M., Solling T.I., Giles M., McCulloch I., Sirringhaus H. (2004). Enhanced mobility of poly(3-hexylthiophene) transistors by spin-coating from high-boiling-point solvents. Chem. Mater..

[5] Ma W., Yang C., Gong X., Lee K., Heeger A.J. (2005). Thermally stable, efficient polymer solar cells with nanoscale control of the interpenetrating network morphology. Adv. Funct. Mater..

[6] Yu H.Z., Peng J.B. (2008). Annealed treatment effect in poly(3-hexylthiophene): Methanofullerene solar cells. Chin. Phys. Lett..

[7] Wu Z., Petzold A., Henze T., Thurn-Albrecht T., Lohwasser R.H., Sommer M., Thelakkat M. (2010). Temperature and molecular weight dependent hierarchical equilibrium structures in semiconducting poly(3-hexylthiophene). Macromolecules.

[8] Cho S., Lee K., Yuen J., Wang G.M., Moses D., Heeger A.J., Surin M., Lazzaroni R. (2006). Thermal annealing-induced enhancement of the field-effect mobility of regioregular poly(3-hexylthiophene) films. J. Appl. Phys..

[9] Yi H.-L., Hua C.-C. (2019). Peculiar aggregation features in poly(3-hexylthiophene)/chlorobenzene solutions. Macromolecules.

[10] Brown P.J., Sirringhaus H., Harrison M., Shkunov M., Friend R.H. (2001). Optical spectroscopy of field-induced charge in self-organized high mobility poly(3-hexylthiophene). Phys. Rev. B.

[11] Brinkmann M., Wittmann J.-C. (2006). Orientation of regioregular poly(3-hexylthiophene) by directional solidification: A simple method to reveal the semicrystalline structure of a conjugated polymer. Adv. Mater..

[12] Yang H., Shin T.J., Yang L., Cho K., Ryu C.Y., Bao Z. (2005). Effect of mesoscale crystalline structure on the field-effect mobility of regioregular poly(3-hexyl thiophene) in thin-film transistors. Adv. Funct. Mater..

[13] Root S.E., Savagatrup S., Printz A.D., Rodriquez D., Lipomi D.J. (2017). Mechanical properties of organic semiconductors for stretchable, highly flexible, and mechanically robust electronics. Chem. Rev..

[14] Ho V., Boudouris B.W., Segalman R.A. (2010). Tuning polythiophene crystallization through systematic side chain functionalization. Macromolecules.

[15] Li D., Xia Y. (2004). Direct fabrication of composite and ceramic hollow nanofibers by electrospinning. Nano Letters.

[16] Babel A., Li D., Xia Y.N., Jenekhe S.A. (2005). Electrospun nanofibers of blends of conjugated polymers: Morphology, optical properties, and field-effect transistors. Macromolecules.

[17] Chen J.-Y., Wu H.-C., Chiu Y.-C., Lin C.-J., Tung S.-H., Chen W.-C. (2015). Electrospun poly(3-hexylthiophene) nanofibers with highly extended and oriented chains through secondary electric field for high-performance field-effect transistors. Adv. Electron. Mater..

[18] Chen J.-Y., Chiu Y.-C., Shih C.-C., Wu W.-C., Chen W.-C. (2015). Electrospun nanofibers with dual plasmonic-enhanced luminescent solar concentrator effects for high-performance organic photovoltaic cells. J. Mater. Chem. A.

[19] Chen J.-Y., Wu H.-C., Chiu Y.-C., Chen W.-C. (2014). Plasmon-enhanced polymer photovoltaic device performance using different patterned Ag/PVP electrospun nanofibers. Adv. Energy Mater..

[20] Tsai P.-C., Chen J.-Y., Ercan E., Chueh C.-C., Tung S.-H., Chen W.-C. (2018). Uniform luminous perovskite nanofibers with color-tunability and improved stability prepared by one-step core/shell electrospinning. Small.

[21] Kuo C.-C., Wang C.-T., Chen W.-C. (2008). Highly-aligned electrospun luminescent nanofibers prepared from polyfluorene/PMMA blends: Fabrication, morphology, photophysical properties and sensory applications. Macromol. Mater. Eng..

[22] Li D., Wang Y., Xia Y. (2003). Electrospinning of polymeric and ceramic nanofibers as uniaxially aligned arrays. Nano Lett..

[23] Li D., Wang Y., Xia Y. (2004). Electrospinning nanofibers as uniaxially aligned arrays and layer-by-layer stacked films. Adv. Mater..

[24] Wen Y.H., Lin P.C., Hua C.C., Chen S.A. (2011). Dynamic structure factor for large aggregate clusters with internal motions: A self-consistent light-scattering study on conjugated polymer solutions. J. Phys. Chem. B.

[25] Guo R.H., Hsu C.H., Hua C.C., Chen S.A. (2015). Colloidal aggregate and gel incubated by amorphous conjugated polymer in hybrid-solvent medium. J. Phys. Chem. B.

[26] Yi H.L., Wu C.H., Wang C.I., Hua C.C. (2017). Solvent-regulated mesoscale aggregation properties of dilute PBTTT-C_14_ solutions. Macromolecules.

[27] Liu H.Q., Reccius C.H., Craighead H.G. (2005). Single electrospun regioregular poly(3-hexylthiophene) nanofiber field-effect transistor. Appl. Phys. Lett..

[28] Liu J., Shao S., Wang H., Zhao K., Xue L., Gao X., Xie Z., Han Y. (2010). The mechanisms for introduction of n-dodecylthiol to modify the P3HT/PCBM morphology. Org. Electron..

[29] Xue L., Yu X., Han Y. (2011). Different structures and crystallinities of poly(3-hexylthiophene) films prepared from aged solutions. Colloids Surf. A.

[30] Berson S., De Bettignies R., Bailly S., Guillerez S. (2007). Poly(3-hexylthiophene) Fibers for Photovoltaic Applications. Adv. Funct. Mater..

[31] Zhang J., Wang C., Chen W., Wu J., Zhang Q. (2015). Fabrication and physical properties of self-assembled ultralong polymer/small molecule hybrid microstructures. RSC Adv..

[32] Hagler T.W., Pakbaz K., Voss K.F., Heeger A.J. (1991). Enhanced order and electronic delocalization in conjugated polymers oriented by gel processing in polyethylene. Phys. Rev. B.

[33] Nguyen T.-Q., Wu J., Doan V., Schwartz B.J., Tolbert S.H. (2000). Control of energy transfer in oriented conjugated polymer-mesoporous silica composites. Science.

[34] Chen J.-Y., Kuo C.-C., Lai C.-S., Chen W.-C., Chen H.-L. (2011). Manipulation on the morphology and electrical properties of aligned electrospun nanofibers of poly(3-hexylthiophene) for field-effect transistor applications. Macromolecules.

