# Supplementary material for: Solvent Effects on Morphology and Electrical Properties of Poly(3-hexylthiophene) Electrospun Nanofibers
Source: Polymers (Basel). 2019 Sep 14;11(9):1501. doi: 10.3390/polym11091501 (PMC6780587; doi:10.3390/polym11091501)
Supplement: Supplementary file 1 [file polymers-11-01501-s001.pdf]

Table S1. Physical properties of three solvents used in this study and those of the P3HT solutions.

Solvent	CF	CB	TCB
Viscosity (cp)	0.45	0.91	1.93
Boiling point (^{o}C)	61.20	131.70	214.00
Solubility parameter (cal cm^{-3})$^{1/2}$	9.50	9.30	9.90
Dielectric constant (-)	4.81	4.62/5.62	2.24
Conductivity (S cm^{-1})	1×10^{-10}	7.0×10^{-11}	1.62×10^{-4}
Surface tension (dyne cm^{-1})	26.53	33.28	39.10
**P3HT Solution**	**CF**	**CB**	**TCB**
Viscosity (cp)	6.22	5.40	21.00
Relative viscosity (-)	13.82	5.91	10.88

Table S2. State-of-the-art electrical performances of pure P3HT electrospun nanofiber-based organic field-effect transistors.

Reference	Method	Core	Shell	Thermal annealing temperature	Mobility (cm^2 V^{-1} s^{-1})
Synth. Met., **2005**, 151, 275.	single capillary electrospinning	P3HT in chloroform	N/A	N/A	4.00×10^{-4}
J. Mater. Chem., **2009**, 19, 743.	core-shell electrospinning	P3HT in chloroform	chloroform	N/A	1.70×10^{-2}
Macromolecules, **2011**, 44, 2883.	core-shell electrospinning	P3HT in chlorobenzene	PMMA in chlorobenzene	100 ^{o}C	1.92×10^{-1}
Adv. Electron. Mater. **2015**, 1: 1400028.	core-shell electrospinning and secondary electric field	P3HT in chloroform	PEO^{a} in chlorobenzene	N/A	1.62×10^{-1}
**This work**	core-shell electrospinning	P3HT in chloroform	PMMA in chlorobenzene	100 ^{o}C	3.57×10^{-1}

^{a}Poly(ethylene oxide) (PEO)
